# Psychometric properties of lift and carry test in assessing people with stroke

**DOI:** 10.3389/fneur.2024.1379536

**Published:** 2024-08-22

**Authors:** Peiming Chen, Mimi M. Y Tse, Shamay S.M. Ng, Leo C. M. Ho, Anthony T. C. Kwok, Sam C. Y. Lam, Tai Wa Liu, Thomson W. L. Wong, Billy C. L. So, Cynthia Y. Y. Lai

**Affiliations:** ^1^Department of Rehabilitation Sciences, The Hong Kong Polytechnic University, Kowloon, Hong Kong SAR, China; ^2^Research Centre for Chinese Medicine Innovation, The Hong Kong Polytechnic University, Kowloon, Hong Kong SAR, China; ^3^School of Nursing and Health Studies, Hong Kong Metropolitan University, Hong Kong, Hong Kong SAR, China

**Keywords:** stroke, upper limb, lower limb, assessment, advance motor function

## Abstract

**Objective:**

To investigate the psychometric properties of the Lift and Carry Test (LCT) time in people with stroke.

**Design:**

Cross-sectional design.

**Setting:**

University based neurorehabilitation laboratory.

**Participants:**

Twenty-four people with stroke and 24 healthy controls.

**Outcome measures:**

Lift and Carry Test (LCT), Fugl-Meyer Assessment of upper extremity and lower extremity, ankle dorsiflexor and plantarflexor muscle strength, Berg Balance Scale (BBS), Timed Up and Go (TUG) and Community Integration Measure.

**Results:**

The mean LCT time (29.70s) in people with stroke was more than double of that in healthy controls (13.70s). The LCT showed excellent intra-rater, inter-rater and test–retest reliability [intraclass correlation coefficient (ICC) = 0.943–1.000]. The LCT times demonstrated a significant negative correlation with the BBS score (r_s_ = −0.771) and significant positive correlations with the TUG times (r_s_ = 0.933). There was no significant correlation between LCT times and FMA score (*p* > 0.05). An optimal cut-off LCT time of 15.48 s (sensitivity = 95.8%, specificity = 87.5%) was identified to differentiate between people with stroke and healthy controls (area under the curve = 0.957).

**Conclusion:**

LCT is an excellent clinical test for examining advanced functional ability in people with stroke and distinguishing people with stroke from healthy controls.

## 1 Introduction

Impaired performance in activities of daily living (ADL), especially lifting and carrying tasks, is common in community-dwelling stroke survivors. Such impairment highly constrains a person’s ability to perform daily tasks, such as household routines and shopping in supermarkets. By improving the functional performance of the upper and lower limbs, the associated negative impacts in daily life can be reduced, which is also the main goal of stroke rehabilitation (1). However, there is no comprehensive assessment tool available to measure the performance of ADL with integrated lifting and carrying components in people with stroke.

**Figure 1:**
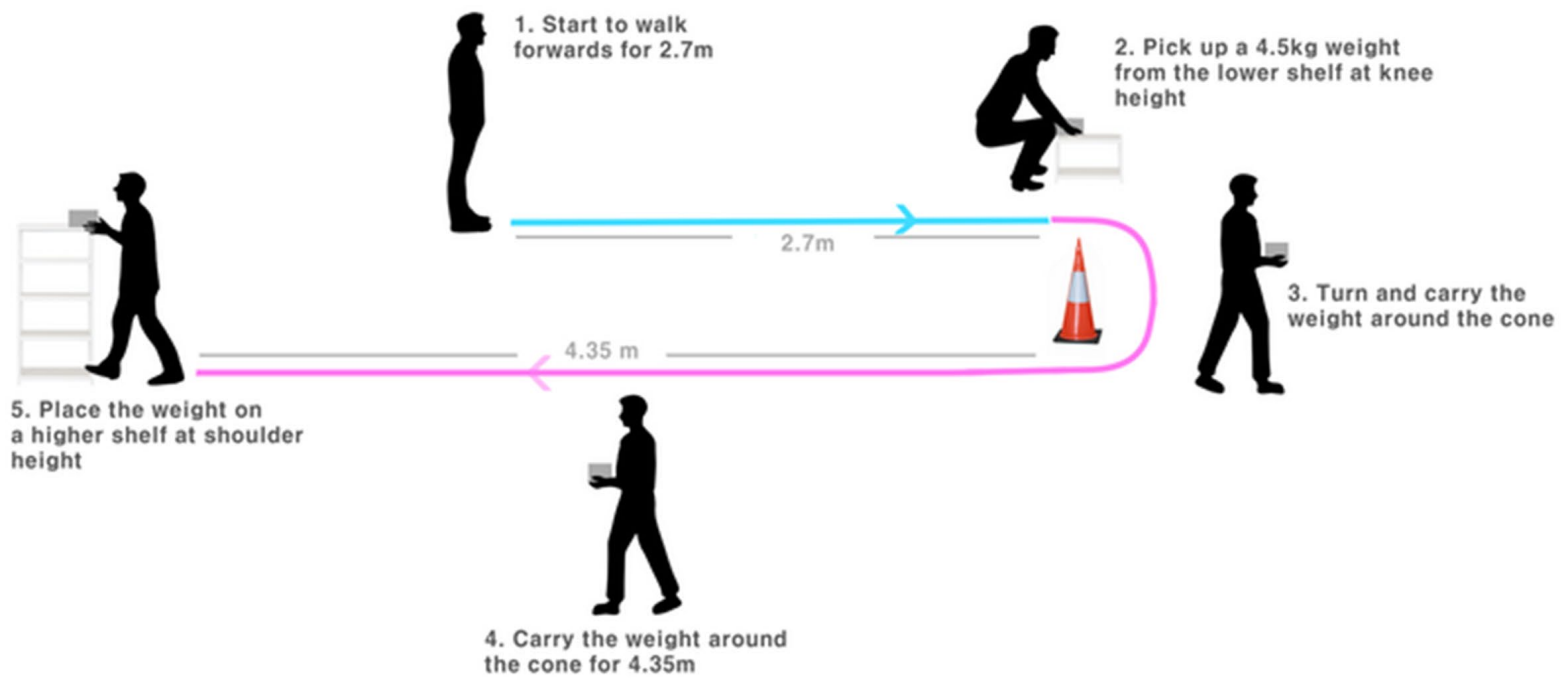
Assessment procedure of LCT.

Lift and Carry Test (LCT) may be useful for evaluating comprehensive ability to perform ADL, which was first developed in 1995 to assess people with knee osteoarthritis (OA), comprises tasks related to walking ability, upper and lower limb function, strength, balance and cognitive function into a single measure (2). The subject is instructed to walk 2.7 m to a set of shelves and lift a 4.5-kg weight from the lower shelf (around knee height); then, they must turn and carry the weight while walking 4.35 m around a cone, return to the shelves, and place the weight on the upper shelf (around shoulder height) as quickly as possible (Figure 1) (2). Previous study showed that LCT completion time had significant negative correlation with tolerance time on the treadmill during modified Naughton treadmill protocol (*r* = −0.40), peak oxygen consumption (*r* = −0.38) and the knee strength (*r* = −0.58) in people with knee OA (3).

In comparison to individuals with unilateral knee osteoarthritis (OA), people who have had a stroke may exhibit even poorer motor function due to hemiparesis affecting their affected upper and lower limbs. This can lead to difficulties in performing common components of daily activities, such as grasping, carrying weight, and walking. As satisfactory level of motor and cognitive functions is required to complete the sequential tasks of LCT (walk and lift weight with both hands from the lower shelf; then, turn and carry the weight while walking around a cone, return to the shelves, and place the weight on the upper shelf), the LCT could be a holistic outcome measure to assess the multiple components, which included the limb motor functional of the affected side, walking ability, balance function and the cognitive function (e.g., executive function), can simulate the ADL in real-world situation (e.g., carrying object when shopping). It can provide insights for designing rehabilitative interventions for community-dwelling people with stroke. However, its psychometric properties have not been investigated in a sample of community-dwelling stroke survivors.

In order to fill the research gap, this study aimed to investigate the psychometric properties of LCT in people with stroke. Many well-developed and highly reliable tools are available for evaluating specific performance attributes in stroke survivors; these include Fugl-Meyer Assessment (FMA), Berg Balance Scale (BBS) and Timed Up and Go Test (TUG), which, respectively, assess motor control of the affected side, balance and mobility (4–6). These reliable tools provide valuable reference for investigating the reliability and validity of LCT.

The medical profession could benefit from a reliable and valid measurement of sequential and advanced functional mobility during ADL in community-dwelling stroke survivors. Therefore, the aim of this study was to investigate: (i) the intra-rater, inter-rater and test–retest reliabilities of LCT time in people with stroke; (ii) the correlations of LCT time with stroke-specific impairment outcome measures, including FMA scores, ankle dorsiflexion and plantarflexion muscle strength, BBS score, TUG time and Community Integration Measure (CIM) score; and (iii) the minimal detectable change (MDC) in LCT time. The study also aimed to (iv) compare LCT time between people with stroke and healthy controls and (v) identify a cut-off LCT time to distinguish performance between these 2 groups.

## 2 Materials and methods

### 2.1 Study design

This was a cross-sectional study approved by the local ethics committee (ethical approval number: HSEARS20160202006). The study objectives and assessment procedures were clearly explained to the participants, who provided written informed consent to participate before the beginning of the study. This study was conducted according to the guidelines of the Declaration of Helsinki.

### 2.2 Sample size calculation

Although no study has investigated the reliability of LCT in people with stroke, this test was shown to have excellent test–retest reliability [intra-class correlation (ICC) = 0.92] in people with knee OA (3). Assuming an ICC cut-off value of 0.75 for assessing test–retest reliability in stroke survivors, a sample size of at least 21 subjects would be required to achieve 80% power to detect an ICC of 0.9, with an ICC of 0.8 as the null hypothesis and a significance level of 0.05. The sample size was estimated using an online calculator (7).

Additionally, no study has investigated the correlations between LCT time and stroke-specific outcome measures in people with stroke. Assuming that LCT time is significantly and moderately strongly correlated (ρ = 0.5) with the selected stroke-specific outcome measures, a sample size of 21 subjects would be required to achieve 80% power and a significance level of 0.05. The sample size was estimated using G∗Power software, version 3.1.9.7 (Franz Faul, University of Kiel, Kiel, Germany).

The estimations indicated that a minimal sample size of 22 would be required to meet the study objectives. To increase the power of this study, 24 subjects with stroke were recruited for assessment of the psychometric properties of LCT.

### 2.3 Participants

**Figure 2:**
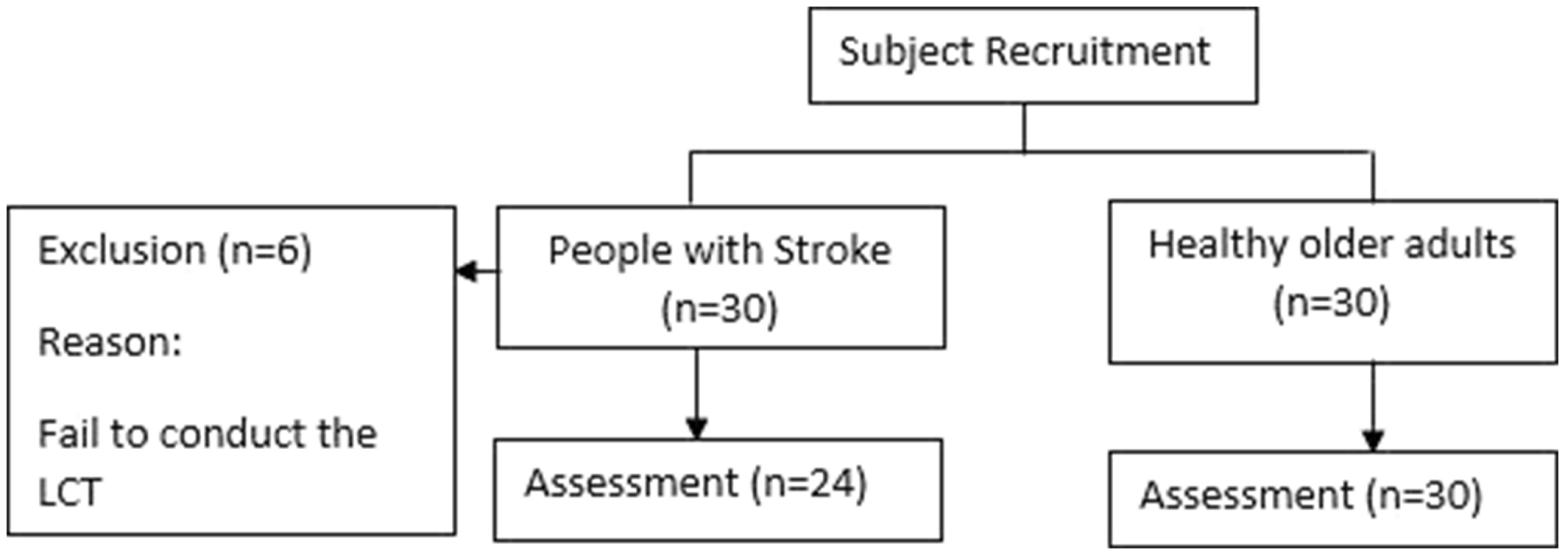
The flowchart of subject recruitment.

Thirty subjects of stroke and 30 healthy older adults were recruited from the community via flyer. Six subjects with stroke failed to conduct the LCT. Thus, 24 subjects of stroke were included in our study (Figure 2). Participants were included if they (i) were aged 55 years or older; (ii) had a post-stroke duration>6 months; (iii) were medically stable; (iv) could walk 10 m without physical assistance from another person, with or without walking aids; and (v) had an Abbreviated Mental Test score ≥ 7 (8). Potential participants with any neurological or cardiovascular diseases that might influenced the assessment were excluded, such as: Parkinson’s disease, Alzheimer’s disease, Multiple Sclerosis and so on.

Twenty-four healthy older adults (3 men, 21 women) aged 55 years or older were recruited as controls.

### 2.4 Testing procedures

**Figure 3:**
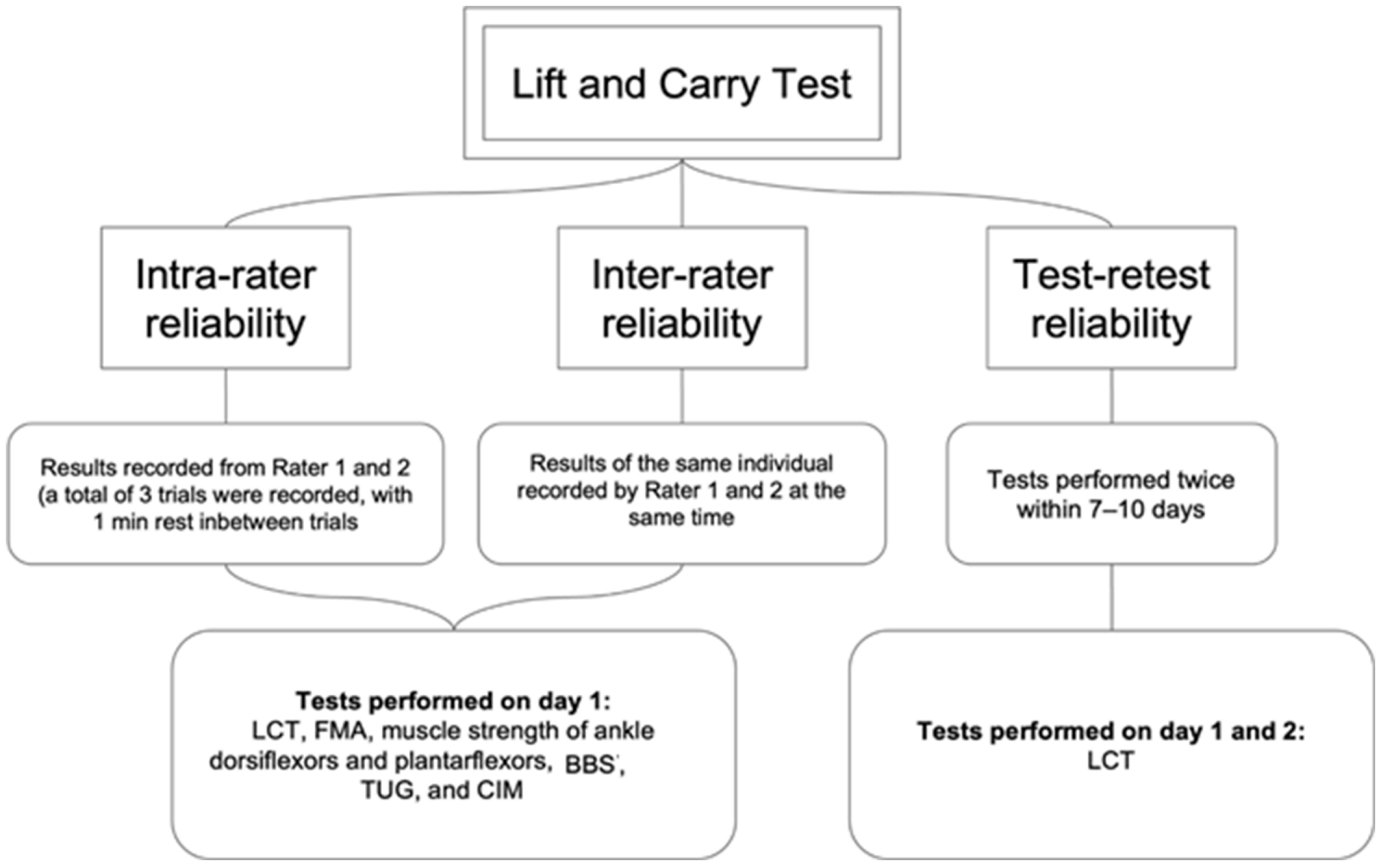
Testing procedures for investigating intra-rater, inter-rater and test-retest reliability of LCT.

To investigate the reliability of LCT in people with stroke, two LCT trials, each comprising one practice and 3 timed tests, were conducted 7 days apart to minimize the learning effect and natural recovery among the participants (Figure 3). Two raters, A and B, were trained at least 2 weeks prior to the start of study. LCT times were measured by both raters simultaneously, using digital stopwatches. The mean time of the 3 timed trials was recorded.

The healthy controls completed the LCT on day 1. The LCT cut-off score for people with stroke was determined.

### 2.5 Outcome measures

#### 2.5.1 Lift and carry test

LCT is used to assess advanced functional mobility (3). It comprises components of different functional tasks, including walking, turning, picking up objects and squatting. The participant is instructed to walk 2.7 m to a set of shelves and lift a 4.5-kg weight with both hands from the lower shelf (around knee height); then, they must turn and carry the weight while walking 4.35 m around a cone, return to the shelves, and place the weight on the upper shelf (around shoulder height) as quickly as possible (Figure 1) (2). In this study, LCT times were recorded independently by 2 well-trained raters. The participants performed 1 practice and 3 timed trials on day 1, with a rest interval of at least 2 min between each. The participants were asked to repeat the same on day 2, which occurred 7 days after day 1. It has been shown good test–retest reliability (ICC = 0.92) in people with bilateral or unilateral knee OA (3).

#### 2.5.2 Fugl-Meyer assessment

FMA is used to assess the motor recovery of the upper and lower extremities of people with stroke, which includes motor control, reflexes and coordination of the upper and lower extremities (9). The upper and lower extremity assessments comprise 33 and 17 items, respectively, and each item is scored on a 3-point scale (0–2). The maximum total score is 100, with maximum scores of 66 and 34 for the assessments of the upper and lower extremities, respectively. It has been shown excellent intra-rater (ICC = 0.96–0.99), inter-rater (ICC = 0.89–0.99) and test–retest reliabilities (ICC = 0.87–0.97) in people with stroke (10, 11).

#### 2.5.3 Muscle strength of the affected and unaffected ankle dorsiflexors and plantarflexors

The ankle dorsiflexor and plantarflexor muscle strength is measured using Lafayette Manual Muscle Testing System Model-01165A (Lafayette Instrument Company, Lafayette, IN, United States). In the beginning, the subject was in the supine position. The ankle was placed in neutral position and knee was placed in the 0 degree flexion. The dynamometer was placed over the first to fifth metatarsal bones anteriorly or posteriorly to test the dorsiflexors or plantarflexors, respectively. Following 1 practice trial, the participants were asked to perform a maximum isometric voluntary contraction (MIVC) for 3 s on both the affected and unaffected sides. The average of 3 trials was recorded for each muscle group. It has been shown excellent test–retest reliability for ankle plantarflexion (ICC = 0.92–0.97) and ankle dorsiflexion (ICC = 0.82–0.95) in people with stroke (12).

#### 2.5.4 Berg balance scale

BBS is used to assess functional balance and mobility in older adults (13). It comprises 14 functional tasks that require individuals to maintain balance in different positions. Each task is graded on a 5-point scale (0–4); the maximum total score is 56, and a higher score indicates better balance. It has been shown excellent inter-rater (ICC = 0.993) and test–retest reliabilities (ICC = 0.99) in people with stroke (13, 14).

#### 2.5.5 Timed up and go test

TUG is used to assess functional mobility (6). In this study, the participants were required to perform 3 consecutive tasks: stand up from a chair, walk 3 m straight ahead, turn 180 degrees, walk back and sit down on the same chair. It has been shown excellent inter-rater and intra-rater reliabilities (ICC = 0.99) and test–retest reliability (ICC = 0.95) in people with strokes.

#### 2.5.6 Community integration measure

CIM is a 10-item measure used to assess the extent of community integration (15). The questions address assimilation, support, occupation and independent living, in accordance with the theoretical model of community integration (16). Each item is scored on a 5-point scale (1–5), with a maximum score of 50; a higher score indicates better community integration. It has been shown good internal consistency and reliability (Cronbach’s alpha = 0.87) in people with stroke (17).

### 2.6 Statistical analysis

Data analysis was conducted using SPSS software, version 26.0 (IBM Corporation, Armonk, NY, United States). The normality of the data and the homogeneity of variances were checked using the Kolmogorov–Smirnov Normality and Levene’s tests, respectively. Demographic data are summarized using mean difference (MD) and standard deviation (SD). The between-group differences of the baseline characteristic among the stroke patients and healthy control were analyzed using independent t-tests for parametric data and the chi-square and Mann–Whitney U-tests for non-parametric data (18).

A two-way mixed-effect model (ICC_3,1_) was used for intra-rater and test–retest reliabilities, as the tests involved 2 independent raters, each of whom recorded 3 trials for each participant. ICC_3,2_ was used for inter-rater reliability as the mean of 3 measurements recorded by a single rater on the same day was compared with the mean of the measurements recorded by the other rater (18). The strength of ICC determined using a general guideline wherein values of <0.5, 0.5–0.75, >0.75–0.9, and > 0.9 indicate poor, moderate, good and excellent reliability, respectively (19). Pearson correlation coefficients and Spearman’s rho is used to examine the correlation between LCT time and the other outcomes, as proper. The *p*-value, which indicates significance, was adjusted to 0.0125 (0.05/4) after Bonferroni adjustment for 4 outcome measures which commonly used to assess different domains of function in people with stroke, which included FMA, BBS, TUG and CIM. These outcome measures were chosen because FMA is a stroke-specific assessment related to lifting and carrying tasks (4, 9, 11), and because strong research evidence supports the excellent reliability of BBS, TUG and CIM, which are also related to walking tasks, in people with stroke (5, 6, 13, 16, 17, 20–22). The secondary outcome measures were ankle plantarflexor and dorsiflexor muscle strength.

The MDC at a 95% confidence level were calculated as follows (23):


MDC=1.96×SEM×√2

SEM is the standard error of measurement of LCT time and was calculated as follows:


$$SEM=S\times \surd \left(1-r\right)\text{,}$$

where *S* is the pooled standard deviation of LCT time, and *r* is the reliability coefficient.

The optimal LCT time cut-offs for differentiating between people with stroke and healthy controls were determined using receiver operating characteristics (ROC) curves. The cut-offs were calculated using Youden’s index to identify the trade-off point between the highest sensitivity and specificity. The area under the curve (AUC) was used to calculate discrimination accuracy.

## 3 Results

The demographic information was shown in Table 1. In this study, the mean post-stroke duration was 3.9 (3.5) years. The mean FMA-UE score was 39.00 (16.65), which indicated moderate level of upper limb motor control (24). The mean FMA-LE score was 24.88 (6.52), which indicated good lower limb motor control (25).

**Table 1:** Demographics of the individuals with stroke and the healthy control.

	Stroke (*n* = 24)	Healthy (*n* = 24)	*p*-value
Age (y)	62.9 (5.9)	59.6 (5.3)	0.143
Sex (M/F)	13/11	3/21	0.002*
Height (cm)	161.5 (9.6)	156.3 (8.7)	0.063
Weight (kg)	63.2 (11.5)	56.6 (9.1)	0.035*
Body mass index (kg/m^2^)	24.2 (3.8)	23.3 (3.9)	0.407
Paretic side (L/R)	10/14	NA	NA
Post-stroke duration (years)	3.9 (3.5)	NA	NA
Type of Stroke (I/H)	15/9	NA	NA

Values are mean (SD) or as otherwise noted. F, female; M, male; L, left; R, right; I, Ischemia; H, Hemorrhage. *Indicates a significant difference at the *p* ≤ 0.05 level of confidence.

The mean LCT times of people with stroke and healthy controls are listed in Table 2. People with stroke had a significantly longer mean LCT time than healthy controls (29.70 (13.20) s vs. 13.7 0 (2.01) s, *p* < 0.001). The mean values of the other outcome measures are also listed in Table 2.

**Table 2:** Outcome measure of people with stroke and healthy control.

Parameters	Individuals with stroke (*n* = 24)		Health (*n* = 24) Mean (SD)	*p* (compared with healthy controls)	*p* (affected side compared with unaffected)
	Affected mean (SD)	Unaffected mean (SD)			
LCT Times (s)	29.70 (13.20)		13.70 (2.01)	<0.001*	
FMA-UE score	39.00 (16.65)				
FMA-LE score	24.88 (6.52)				
Muscle strength					
Ankle Dorsiflexion (kg)	10.90 (5.28)	17.30 (3.88)			<0.0001*
Ankle Plantarflexion (kg)	11.40 (0.97)	16.90 (4.68)			0.003
BBS score	51.58 (3.12)				
TUG times (s)	19.20 (8.60)				
CIM score	41.90 (6.28)				

Mean values were calculated from all the trials from both raters on both days. *Indicates a significant difference at the *p* ≤ 0.05 level of confidence. SD, Standard deviation; FMA-LE, Fugl-Meyer Motor Assessment score of lower extremity; FMA-UE, Fugl-Meyer Motor Assessment score of upper extremity; BBS, Berg Balance Scale; TUG, Timed Up and Go; CIM, Community Integration Measure.

**Figure 4:**
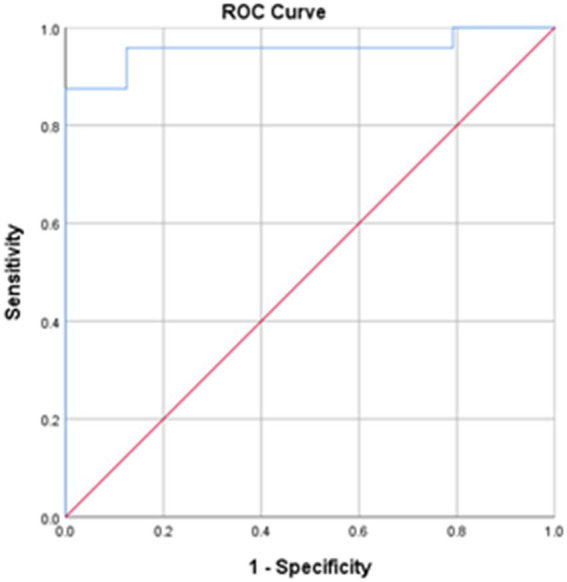
The receiver operating characteristic (ROC) curves for the LCT times. Area under the curve (AUC) = 0.957; sensitivity = 95.8%; specificity = 87.5%; *p < 0.001.

LCT was found to have good to excellent intra-rater, inter-rater and test–retest reliabilities, with respective ICCs of 0.943–0.997, 0.989–1.000 and 0.983–0.995 (Table 3). The MDC of LCT time at the 95% confidence level was 2.32 s. The best cut-off LCT time for differentiating between people with stroke and healthy controls was 15.48 s (AUC = 0.957, sensitivity = 95.8%, specificity = 87.5%, *p* = 0.035, Figure 4).

**Table 3:** Intra-rater, inter-rater and test–retest reliability of the LCT completion times in individual with stroke.

Rater	Day 1	Day 2	Mean of day 1 and day 2
	ICC_3,1_ (95% CI)	ICC_3,1_ (95% CI)	ICC_3,1_ (95% CI)
**Intra-rarer reliability**			
Rater 1	0.985 (0.970–0.993)	0.993 (0.986–0.997)	0.975 (0.943–0.989)
Rater 2	0.985 (0.969–0.993)	0.993 (0.985–0.997)	0.976 (0.944–0.990)
**Inter-rater reliability**			
Rater 1 and 2	0.994 (0.989–0.997)	0.997 (0.995–0.999)	1.000 (0.999–1.000)
**Test–retest reliability**			
Rater 1	0.990 (0.983–0.995)		
Rater 2	0.990 (0.983–0.995)		

95% CI: 95% confidence interval; ICC, intraclass correlation coefficient.

The LCT time was significantly correlated with BBS score (r_s_ = −0.771, *p* < 0.001) and TUG time (r_s_ = 0.933, p < 0.001; Table 4).

**Table 4:** Partial Correlations relating the LCT completion times with indicators of stroke-specific impairment.

	Time	*p*-value
FMA-UE score	−0.132	0.559
FMA-LE score	−0.208	0.352
Muscle strength		
**Ankle dorsiflexors**		
Affected	−0.265	0.234
Unaffected	−0.046	0.838
**Ankle plantarflexors**		
Affected	−0.031	0.890
Unaffected	−0.046	0.838
BBS score	−0.771*	<0.001
TUG times	0.933*	<0.001
CIM score	−0.142	0.529

*Significant correlation after Bonferroni adjustment at a *p*-value of 0.05/4 (*p* ≤ 0.0125). FMA-LE, Fugl-Meyer Motor Assessment score of lower extremity; FMA-UE, Fugl-Meyer Motor Assessment score of upper extremity; BBS, Berg Balance Scale; TUG, Timed Up and Go; CIM, Community Integration Measure.

No significant correlation between LCT time and FMA, muscle strength of ankle plantarflexor and dorsiflexor and CIM score (*p* > 0.05).

## 4 Discussion

### 4.1 Summary

This is the first study to determine that LCT time has excellent intra-rater, inter-rater and test–retest reliabilities and is significantly correlated with other stroke-specific outcome measures. It is also the first study to determine the optimal cut-off LCT time for differentiating performance of LCT between people with stroke and healthy controls.

### 4.2 Reliability of LCT

As noted, LCT exhibited excellent intra-rater, inter-rater and test–retest reliabilities. A study also indicated that LCT has good to excellent test–retest reliability in people with knee OA and hip (ICC = 0.92 at 14 days, ICC = 0.77 at 3 months) (2). This excellent reliability may be attributable to the standardization of the testing procedure and a high level of consistency in various rater and participant aspects. Our raters were well-trained in performing the measurement and strictly followed the formulated test procedures. Clear instructions were delivered to the participants, whose comprehension was ensured. The 7-day test–retest span partly eliminated the practice effect and minimized the cultivation effect on the participants’ performance (26).

### 4.3 Performance of LCT

People with stroke had a nearly two-fold significant longer mean LCT time than did healthy controls. LCT is an assessment of advanced functional activity and assesses complex and coordinated upper and lower limb motor control, as well as sensory and cognitive function. LCT comprises sequential tasks involving walking, bending down, picking up objects and turning (2). Previous studies (27, 28) found that motor sequence learning, which is suggested to be dominated by the working memory (29), was impaired after stroke. Impairments in motor control function and cognitive function following stroke may explain why people with stroke needed a longer time to complete the LCT than healthy controls (30).

In fact, our findings were consistent with those of previous studies on the performance of individual components of LCT (2). People with stroke were found to walk at a slower speed (stroke = 0.88 m/s, healthy = 1.49 m/s) and a shorter distance (stroke = 261.5 m, healthy = 660.1 m) than those of healthy controls on level ground (31), and had difficulties in picking up objects, requiring 50% more time to complete trunk–scapular forward and backward bending than healthy controls (32). Moreover, in one study, people with stroke required 3 more seconds to turn than healthy controls (33). Poor post-stroke muscle strength resulting from reductions in the firing rates and number of fast-twitch fibers; poor motor control, coordination and balance; and spasticity may explain why people with stroke took longer than healthy controls to perform each movement in LCT (2, 34). In addition, sensory deficits can also lead to an increase in LCT time (35). Sensorimotor integration is affected in people with stroke, which may disrupt coordination of the gaze and posture while turning. Such deficits may have led to a large discrepancy in LCT times between the groups in our study.

Apart from motor control, the sequential tasks in the LCT place a cognitive demand on the individual. Studies have demonstrated that reductions in gait and balance in people with stroke during dual tasks were due to cognitive–motor interference (36). Some other studies (27, 28) also supported that motor sequence learning was impaired after stroke, which can affect their ability to perform motor skill with complex sequence of movement. During the LCT, the participants in our study were required to remember to pick up an object from the lower shelf, walk in a fixed sequence while carrying it, and return it to the higher shelf. It is possible that the participants with stroke may have taken longer to complete the test, in part, due to a higher cognitive demand related to the task.

The MDC of the LCT time was 2.32 s. In this study, the actual mean difference in LCT time between stroke survivors and healthy controls, 16.00 s, was much greater than the MDC and thus indicates an actual difference in functional ability rather than a measurement error.

LCT was originally designed to examine the functional limitations of people with knee OA, as people with severe OA experience greater dysfunction when lifting and carrying objects than people with a less severe condition (37). In a comparison with the findings from previous studies, people with stroke in our study were found to require a much longer mean time to complete LCT (29.70s) than people with knee OA (11.0 s). This difference may be attributed to greater dysfunction in people with stroke. In people with knee OA, walking ability is only impeded by musculoskeletal problems in the lower limbs. In contrast, people with stroke are also restricted by a spectrum of stroke-specific deficits, including upper and lower limbs, trunk control and cognition (2, 34, 35).

### 4.4 Correlations of the LCT time with other outcome measures

LCT time was not found to be significantly correlated with FMA total score. FMA was originally designed to assess motor impairment in people with stroke. This assessment of the motor control, reflex and coordination of the upper and lower extremities is performed in static positions, such as lying, sitting or standing (9). In this study, the mean FMA-UE and FMA-LE score was 39.00 (16.65) and 24.88 (6.52), respectively, which indicated moderate to upper limb (24) and lower limb motor control (25) of the affected side. However, LCT is an advanced functional task that places both physical and cognitive demands on the participant. For example, during LCT, participants perform dynamic transitional movements, such as trunk bending and walking, and are required to follow multi-step commands, as mentioned previously (2), and these requirements pose challenges to participants’ memory and cognitive skills. Moreover, there are no limitations on participants’ use of their upper extremities to pick up the 4.5-kg object during the LCT, which means that they can use their unaffected upper extremity. Rather than Solely focusing on the motor performance of the affected side as assessed by FMA, the LCT more likely to consider how people with stroke can modify and adjust their performance of multiple ADL task with the motor function of both the affected and unaffected side. These may explain the lack of correlation between LCT time and FMA total score.

No significant correlation was shown between the ankle muscle strength on either the affected or unaffected side and LCT time. A significant association between lower limb muscle strength, especially the ankle dorsiflexor, and walking speed in the swing phase has been reported. The ankle dorsiflexors are important during the swing phase (38), as they allow for sufficient foot clearance. Indeed, LCT is a sequential advanced functional assessment that places both physical and cognitive demands on the participant. The insignificant correlation between ankle muscle strength and LCT time may indicate that the cognitive function may play a more important role than the ankle muscle strength in the LCT completion time. In addition, the LCT is a multiple task required the coordination of both the affected and unaffected limbs, which is far more complicate than the pure muscle contraction in the unilateral muscle testing. It is reasonable that there was no significant correlation found between LCT times and muscle strength. Further complementary study should include the assessment tool of cognitive function, so that to determine the role of cognitive function in the LCT completion time.

LCT time was found to have a significant negative correlation with BBS score. BBS includes tasks such as turning (items 10, 11) and picking up objects from the floor (item 9), which are highly related to several skill components in LCT (e.g., bending to pick up a 4.5-kg weight and turning). These similarities may explain the observed correlation between LCT time and BBS score.

No significant correlation was observed between LCT time and CIM score in this study. There are several reasons which may contribute to this insignificant relationship between LCT time and CIM score. First, the measurement domains between LCT and CIM is different. The CIM is a subjective measurement of community integration that considers the self-reported subjective feelings of participants (2) while the LCT is an objective measurement of sequential motor tasks in a laboratory setting (15), which may not reflect the subjective feelings of participants in real-life situations. Second, previous studies (39, 40) have proven that motor function alone is insufficient in predicting the community reintegration of people with stroke. Other factors, including poststroke depression, personal perceptions, and stroke recovery, are also important predictors of community reintegration in this population (*r* = 0.499–0.743, *p* < 0.01)(39, 40). Third, 3-factor structure had been identified in CIM for use in stroke survivors living in Hong Kong by Principal Component Analysis, including “relationship and engagement,” “sense of knowing” and “independent living” (17). The LCT is an objective measurement of sequential motor tasks which relates on “independent living,” but not on “relationship and engagement,” “sense of knowing.”

### 4.5 Limitation of LCT

This study has several limitations. First, only LCT time was measured in this study. Although the LCT completion time is easy to obtain and easy-to-administrate, it may not be valid to assess the quality of movement. The quality of the participants’ movement and how they used compensatory strategies to execute the tasks were not examined; therefore, future studies might further investigate the qualitative aspects of LCT. Second, the measure of intra-rater reliability was constrained to 10 min after the first trial. Accordingly, this measure could only reflect excellent test–retest reliability over a short timespan across trials, and therefore, the short-term learning effect may have affected the results. Future studies may consider using a longer test–retest time span. Third, the cut-off score may not be generalizable due to the significant difference in sex ratio between the groups. Studies (41, 42) have reported significant differences in muscle strength and motor performance between male and female participants, and such gender-relate differences may also contribute to the significant difference of the motor function between the stroke and health groups, and eventually lead to the significant difference of the LCT performance. Precaution should be taken before interpreting the result. To enlarge the subject pool to ensure the equivalent gender-ratio between the 2 groups in the future study could help to address this limitation. Fourth, a sample size of 22 was estimated for this study in accordance with previous studies involving people with knee osteoarthritis. The conclusion drawn from our study may only applicable to the subject who meet our inclusion criteria. We may not have sufficient power to confirm whether there was a potential correlation in some of the outcomes. Fifth, the LCT is a multidimensional outcome, which encompasses tasks related to walking ability, upper and lower limb function, strength, balance, and cognitive function. This confluence of diverse factors implies a violation of unidimensionality of the outcome measures, thereby prompting the variation of the latent variable being investigated by LCT completion time in people with stroke. By elucidating the relationship between LCT performance and various clinical measures in the future study, it becomes conceivable to ascertain the specific functional abilities and limitations captured by LCT. Finally, people with stroke were recruited from the community for this study. These participants generally had better walking capability and functional ability, which may have led to selection bias. In summary, future studies aiming to investigate the concurrent and predictive validity of LCT should consider using larger sample sizes and including a more diverse population of people with stroke in terms of walking ability, which will enable generalization of the results to all people with stroke.

## 5 Conclusion

LCT is a reliable and valid clinical tool for assessing the advanced functional performance of ADL involving lifting and carrying tasks. Furthermore, LCT time could be used to differentiate between healthy adults and people with stroke. Therefore, LCT is a feasible clinical tool for assessing the advanced functional performance of people with stroke and ensuring that they reach your motor potential or functional independence.

## Data Availability

The raw data supporting the conclusions of this article will be made available by the authors, without undue reservation.
